# Disulfiram/Copper Combined with Irradiation Induces Immunogenic Cell Death in Melanoma

**DOI:** 10.3390/ijms27020980

**Published:** 2026-01-19

**Authors:** Enwen Wang, Yida Zhang, Lin Jia, Zunwen Lin, Ting Sun, Pan Hu, Kun Wang, Zikun Shang, Wei Guo, Juliann G. Kiang, Xinhui Wang

**Affiliations:** 1 Division of Gastrointestinal and Oncologic Surgery, Department of Surgery, Harvard Medical School, Massachusetts General Hospital, Boston, MA 02114, USA; enwang313@cqu.edu.cn (E.W.); yidazh@126.com (Y.Z.);; 2 Radiation Combined Injury Program, Armed Forces Radiobiology Research Institute, Uniformed Services University of the Health Sciences, Bethesda, MD 20814, USA; juliann.kiang@usuhs.edu

**Keywords:** melanoma, immunogenic cell death, disulfiram/copper, irradiation

## Abstract

Immunogenic cell death (ICD) is a programmed pathway leading to cell death and promotion of immunological responses. Melanoma is resistant to chemotherapy and radiotherapy (RT). Disulfiram (DSF), which forms complexes with copper (Cu), has been shown to induce ICD of many tumor types. Here, we aim to investigate whether DSF/Cu combined with irradiation (IR) can induce ICD and exert anti-cancer effects in melanoma. In vitro experiments, treatment of MV3 and B16F10 melanoma cells with DSF/Cu + IR significantly increased the cellular apoptosis and increased ICD markers: damage-associated molecular pattern molecule (DAMP) exposure and release, including calreticulin cell surface expression, high-mobility group box 1 (HMGB1) release, and decreased intracellular ATP levels. In addition, DSF/Cu combined with IR treatment inhibited tumor growth and enhanced tumor-infiltrating immune cells in the B16F10-bearing C57BL/6 model. Our findings reveal that combining IR with DSF/Cu induces ICD and inhibits tumor growth in melanoma, providing a promising strategy to overcome the inherent resistance of RT in melanoma.

## 1. Introduction

Cutaneous melanoma incidence has increased over the past several decades and remains the most lethal form of cutaneous neoplasm. Standard melanoma treatment options developed over the years include surgery, molecular targeted therapy, radiotherapy (RT) and chemotherapy. Recently, various immunotherapy-based protocols have been shown to have a significant impact on the success of cancer treatments against a variety of tumor types, including melanoma [1]. Immunotherapy, represented by immune checkpoint inhibitors (ICIs), has revolutionized the clinical treatment of melanoma, particularly for advanced or metastatic cases [2,3]. Nevertheless, even when ICI is combined with other strategies, approximately half of patients fail to derive long-lasting benefit [4,5]. This indicates that there is an urgent need to identify new strategies to achieve more effective combined treatments and overcome immune resistance.

Compared to many other types of cancers, melanoma is resistant to chemotherapy and radiotherapy, owing to profound tumor cell plasticity mediated by genetic mutation and activation of alternative mechanisms, and tumor microenvironment (TME) [6]. For example, chemotherapy only provides little therapeutic benefit to patients with advanced melanoma, as proven by a limited response rate of 10–15% to dacarbazine-based chemotherapy [7]. In addition, mutations in the mitogen-activated protein kinase (MAPK) pathway, particularly BRAF mutations, have been identified as key driving genetic aberrations in melanoma [8]. Several targeted therapies, including vemurafenib (a BRAF inhibitor) and trametinib (a MEK inhibitor), have proven effective in patients with melanoma harboring specific mutations. However, acquired resistance is a key obstacle preventing melanoma patients from benefiting from targeted therapies [9]. Apoptosis is an important mechanism for inducing tumor cell death by chemotherapy and radiotherapy. However, melanoma often evades apoptosis by up-regulating the expression of anti-apoptotic proteins, such as melanoma inhibitor of apoptosis protein (ML-IAP) [10]. For optimum melanoma patient outcomes, RT needs to be integrated with other systemic therapies [11]. ICIs have significantly prolonged the progression-free survival and overall survival of melanoma patients [12,13,14,15,16]. Currently, clinical evidence suggests that the potential of enhancing the abscopal effect of RT on the treatment of malignant melanoma by combining it with immunotherapeutic agents, such as the ICIs including PD-1/PD-L1 and CTLA-4 [17]. However, treatment outcomes remain unsatisfactory for many patients with advanced melanoma, as a substantial proportion experience disease progression following ICI-based immunotherapy.

Disulfiram (DSF), an FDA-approved and safe drug for treating alcoholism, has been shown to exert great potential as an anticancer drug [18]. Within cells, DSF is converted to diethyldithiocarbamate, which binds to copper ion (Cu^2+^) to form the complex (DSF/Cu) [19,20,21]. Compelling evidence suggests that DSF/Cu manifested more serious toxicity against cancer cells than DSF alone. For instance, DSF/Cu induces nuclear accumulation of nuclear protein localization protein 4(NPL4), leading to disruption of p97–NPL4 function disruption of p97–NPL4 function [22], inhibits nuclear factor-κB (NF-κB) pathway [19,23], triggers endoplasmic reticulum (ER) stress by activating the IRE1α-XBP1 axis [24], and induces immunogenic cell death (ICD) in both differentiated and irradiation (IR)-resistant cancer stem cell (CSC) populations [25,26]. DSF/Cu has been demonstrated to induce tumor cell death in various malignancies [27,28,29,30]. For instance, DSF/Cu triggers autophagic cell death in colorectal cancer (CRC) cells by upregulating expression of UNC-51-like kinase 1 (ULK1), a central regulator in autophagy [27]. In addition, DSF/Cu displays antitumor activity against nasopharyngeal cancer cells through stimulating ROS/MAPK and ferroptosis pathways [28]. Its repurposing for cancer therapy was initially considered to be due to DSF/Cu’s ability to block the neutralization of the genotoxic aldehydes generated by IR and most cancer chemotherapeutic agents and, as a result, promote tumor cell death. Nowadays, DSF/Cu has been a promising antitumor strategy due to its outstanding anticancer activity and safety.

ICD is a cell death process characterized by upregulated expression of several damage-associated molecular pattern molecules (DAMPs) [31], including cell surface translocation of calreticulin (CRT), increased extracellular ATP and released high-mobility group box 1 (HMGB1). Most importantly, it promotes immune responsiveness. Mechanistically, CRT serves as an “eat me” signal molecule, attracting antigen-presenting cells (APCs) to phagocytize the apoptotic or dead tumor cells. Moreover, ATP acts as a “find me” signal facilitating immune cells to infiltrate into TME [32,33,34,35]. Consequently, ICD promotes subsequent adaptive immune response, enhancing the host anti-tumor immune response [34,36]. It is well known that immune cells eliminate dead cells during infections and injuries, a process that ultimately results in host immune responses. Combining IR with the ICD inducer can enhance anti-tumor immune responses [17]. Recently, we found that combining IR with DSF/Cu led to a more robust level of ICD of differentiated cancer cells and cancer stem cells than was achieved by either method alone in previous studies [25,26,37].

In this study, our objective is to investigate the ability of DSF/Cu + IR to improve the induction of ICD of melanoma in vitro and in vivo experiments. Our findings provide a promising strategy to overcome the inherent resistance of melanoma to conventional therapies.

## 2. Results

### 2.1. DSF/Cu Decreases Viability of Melanoma Cells In Vitro

**Figure 1:**
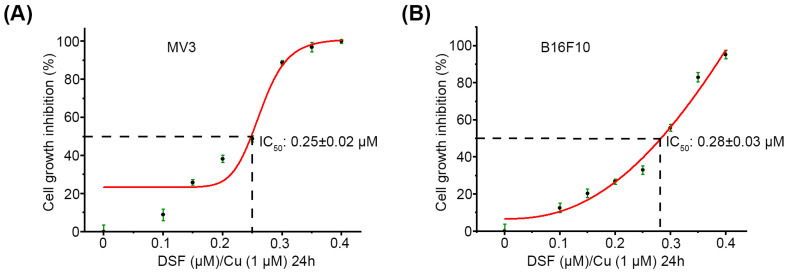
Cell viability of MV3 and B16F10 cells treated with DSF/Cu. After MV3 and B16F10 cells were treated with DSF: 0–0.4 µM, Cu: 1 µM for 24 h. Cell viability was assessed using the MTT assay for MV3 (**A**) and B16F10 cells (**B**). Data were generated in triplicate and are presented as mean ± SD. The R^2^ values of the fitted curves were 0.9683 for MV3 cells and 0.9820 for B16F10 cells.

To examine the effect of DSF/Cu treatment on the viability of melanoma cells in vitro, the MTT assay was applied to evaluate the cell viability of MV3 and B16F10 cells treated with DSF/Cu (DSF: 0–1 µM, Cu: 1 µM). DSF/Cu treatment for 24 h significantly inhibited the viability of MV3 and B16-F10 cells in a dose-dependent manner (Figure 1A,B). In addition, the IC_50_ of DSF/Cu (Cu: 1 µM) for MV3 and B16F10 cells were 0.25 ± 0.02 µM (Figure 1A) and 0.28 ± 0.03 µM (Figure 1B), respectively. These results indicate that DSF/Cu exerts anticancer function in melanoma and provide a basis for the selection of DSF concentration for subsequent cell experiments in vivo.

### 2.2. DSF/Cu + IR Induces Apoptosis of Melanoma Cells In Vitro

**Figure 2:**
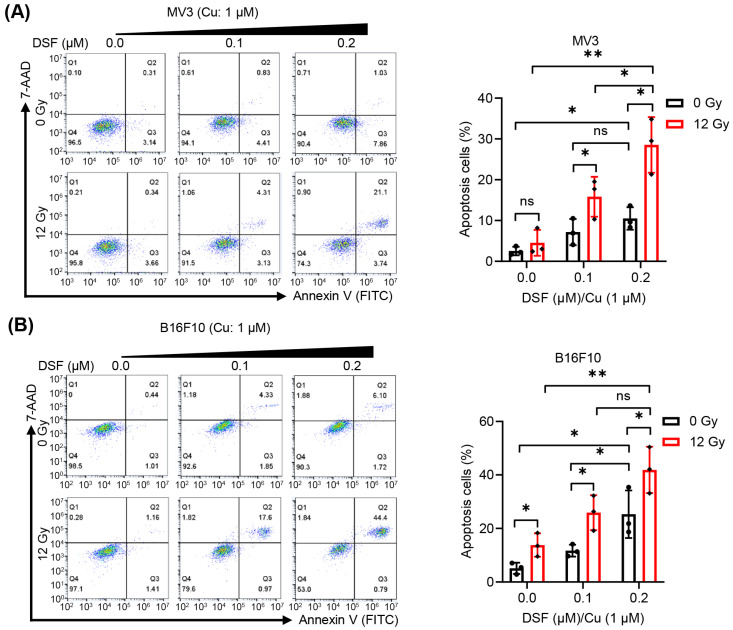
DSF/Cu (Cu: 1 μM) + IR induces apoptosis of MV3 and B16F10 melanoma cells. After MV3 and B16F10 cells were treated with DSF: 0–0.2 µM, Cu: 1 µM and IR (0, 12 Gy) for 24 h, apoptotic cell rate (%) is determined by measuring Annexin V/7-AAD expression using flow cytometry (**A**,**B**). *n* = 3, * *p* < 0.05; ** *p* < 0.01; ns represents no significant difference.

Next, to examine whether DSF/Cu can enhance the ICD and anti-tumor effects induced by IR, we chose low concentrations of DSF (0–0.2 µM)/Cu (Cu: 1 µM) combined with IR (12 Gy) to treat melanoma cells in vitro. Apoptotic MV3 and B16F10 cells were identified by flow cytometry analysis using 7-AAD and Annexin V staining, as shown in Figure 2. Without IR treatment, DSF (0.1 or 0.2 µM)/Cu (Cu: 1 µM) dramatically induces apoptosis of MV3 and B16F10 cells. When combined with IR (12 Gy) treatment, DSF (0.1 or 0.2 µM)/Cu (1 µM) further exacerbated the apoptotic rates of MV3 and B16F10 cells (Figure 2A,B), respectively. These results reveal that DSF/Cu can not only induce apoptosis in melanoma cells but also can enhance the apoptosis induced by IR.

### 2.3. DSF/Cu + IR Induces ICD of Melanoma Cells In Vitro

**Figure 3:**
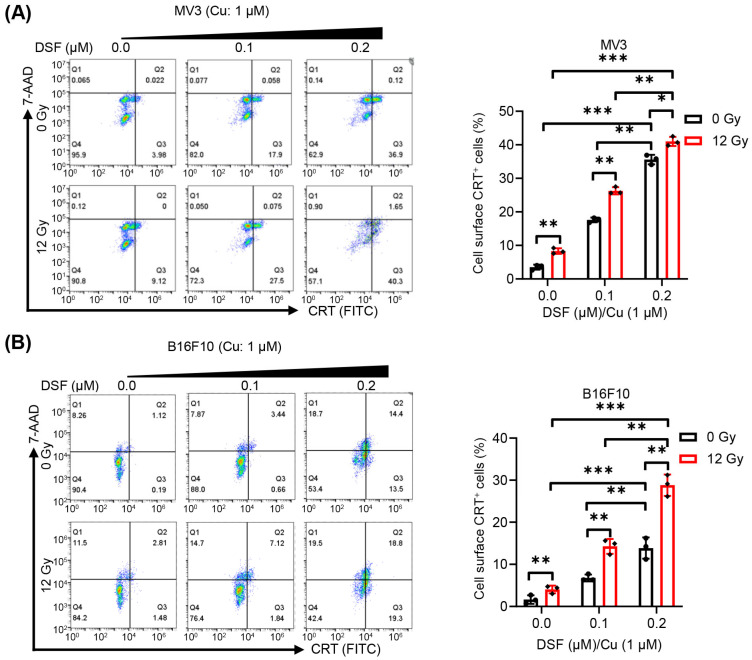
Cell surface detection of CRT by MV3 and B16F10 cells following treatment with DSF/Cu + IR. After treatment with (DSF: 0–0.2 µM, Cu: 1 µM) and IR (0, 12 Gy) for 24 h, Cell surface CRT expression of MV3 and B16F10 cells is determined by flow cytometry (**A**,**B**). *n* = 3, * *p* < 0.05; ** *p* < 0.01; *** *p* < 0.001.

**Figure 4:**
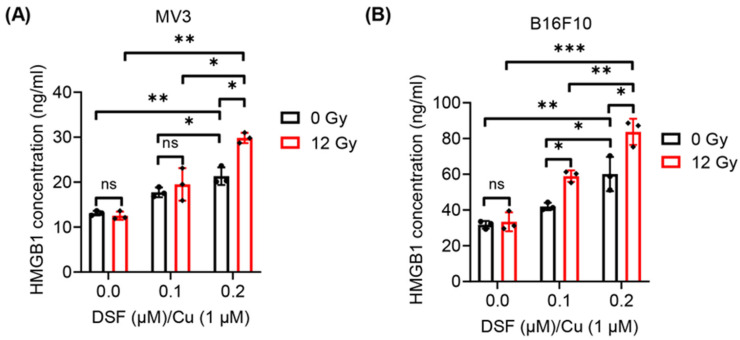
HMGB1 release from MV3 and B16F10 cells following treatment with DSF/Cu + IR. After treatment with DSF: 0–0.2 µM, Cu: 1 µM and IR (0, 12 Gy) for 24 h, HMGB1 release from MV3 and B16F10 cells was determined by ELISA (**A**,**B**). *n* = 3, * *p* < 0.05; ** *p* < 0.01; *** *p* < 0.001; ns represents no significant difference.

**Figure 5:**
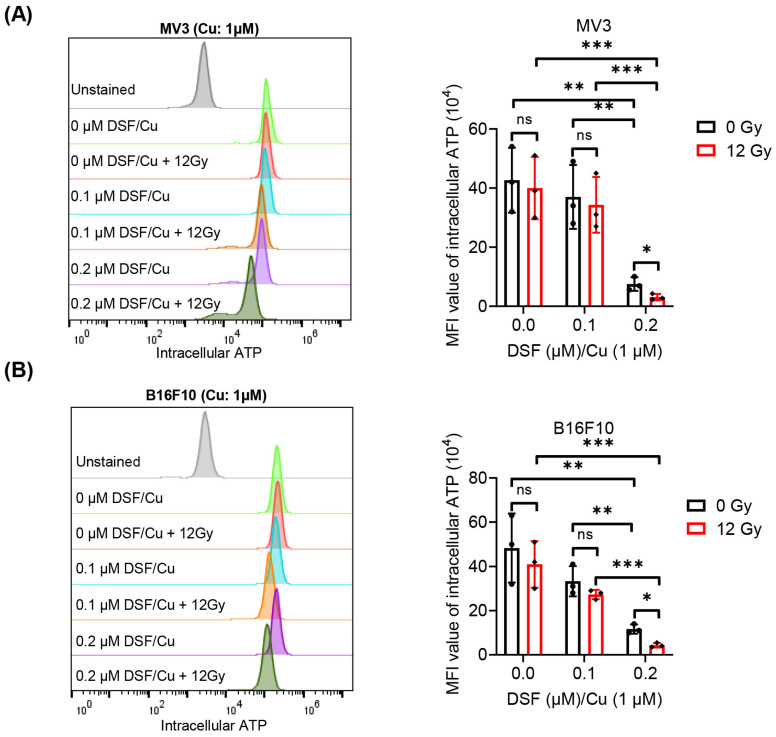
Intracellular ATP levels in MV3 and B16F10 cells following treatment with DSF/Cu + IR. After treatment with DSF: 0–0.2 µM, Cu: 1 µM and IR (0, 12 Gy) for 24 h, Intracellular ATP levels in MV3 and B16F10 cells were determined by flow cytometry (**A**,**B**). *n* = 3, * *p* < 0.05; ** *p* < 0.01; *** *p* < 0.001; ns represents no significant difference.

To assess the ICD induced by DSF/Cu + IR in melanoma cell lines, the exposure and release of DAMPs, such as CRT, ATP and HMGB1, were evaluated. As the concentration of DSF increased and without IR treatment, the translocation of cell surface CRT and the release of HMGB1 in MV3 and B16F10 cells were enhanced under the DSF/Cu (Cu: 1 µM) treatment in a dose-dependent manner (Figure 3 and Figure 4). These results indicate that DSF markedly upregulates DAMPs in melanoma cell lines. In addition, decreased intracellular ATP levels, reflecting increased extracellular ATP release, were observed in DSF (0.2 µM)/Cu (Cu: 1 µM)-treated MV3 and B16F10 cells (Figure 5). Furthermore, treatment of both MV3 and B16F10 cells with DSF/Cu (Cu: 1 µM) combined with IR (12 Gy), in a dose-dependent manner, significantly enhanced the cell-surface CRT expression and the release of HMGB1 (Figure 3 and Figure 4) while decreasing intracellular ATP levels (Figure 5). These results demonstrate that DSF/Cu + IR observably triggers DAMP exposure and release and induces ICD in melanoma cells.

### 2.4. Impact of DSF/Cu + IR on Tumor Growth in the Mouse B16F10 Melanoma Model

**Figure 6:**
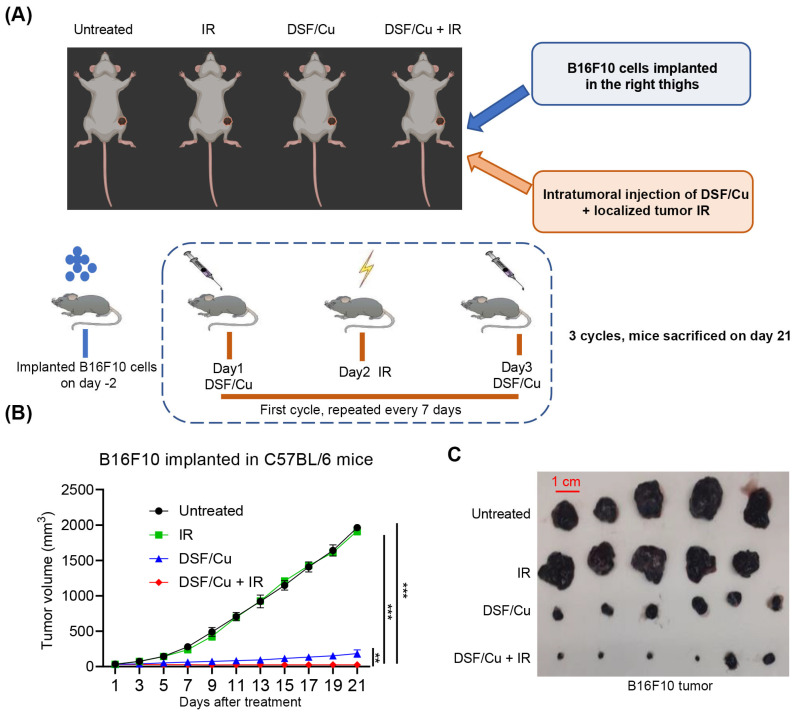
DSF/Cu + IR inhibits growth of B16F10 melanoma in vivo. B16F10 cells were implanted in C57BL/6 mice. Untreated group: mice were left untreated. DSF/Cu group: mice received intratumoral (i.t.) injections of 100 µL PBS containing DSF/Cu (DSF, 0.3 µM; Cu, 1 µM) on days 1 and 3. IR (12 Gy, single dose) group: tumor-localized irradiation (12 Gy) was delivered after the first intratumoral DSF in-jection in each treatment cycle, for a total of three cycles, with the remainder of the body shielded by lead. DSF/Cu + IR group: mice received intratumoral (i.t.) injections of 100 µL PBS containing DSF/Cu (DSF, 0.3 µM; Cu, 1 µM) on days 1 and 3. Tumor-localized IR (12 Gy) was delivered once on day 2. This treatment cycle was repeated three times at 7-day intervals (**A**). Tumor growth was monitored every two days. Tumor growth (**B**), Photos of isolated tumors from each group (**C**) were displayed. *n* ≥ 5, ** *p* < 0.01; *** *p* < 0.001.

Next, we explored whether DSF/Cu + IR could significantly inhibit the growth of melanoma in vivo. Tumor growth in B16F10-bearing C57/BL6 mice was treated with DSF/Cu intratumorally and a single dose of localized tumor IR (12 Gy) (one cycle). Four groups of randomly selected C57BL/6 mice bearing B16F10 tumors—untreated, DSF/Cu, IR, and DSF/Cu + IR—were employed to determine the impact of DSF/Cu + IR on growth of the melanoma in mice. After three cycles of administration, mice were sacrificed on day 21, and tumors were measured (Figure 6A). Treatment of the mice with IR had no noticeable impact on tumor growth in the tumor-bearing mice compared to the untreated group. In contrast, DSF/Cu administration alone significantly inhibited tumor growth compared to the IR and untreated groups (Figure 6B,C). Furthermore, tumor growth was more significantly inhibited in the mice treated with DSF/Cu + IR compared to DSF/Cu alone, indicating that DSF/Cu potentiates inhibitory effects of IR on the growth of melanoma in vivo.

### 2.5. Levels of Distinct Subpopulations of Infiltrating Immune Cells in Tumors

**Figure 7:**
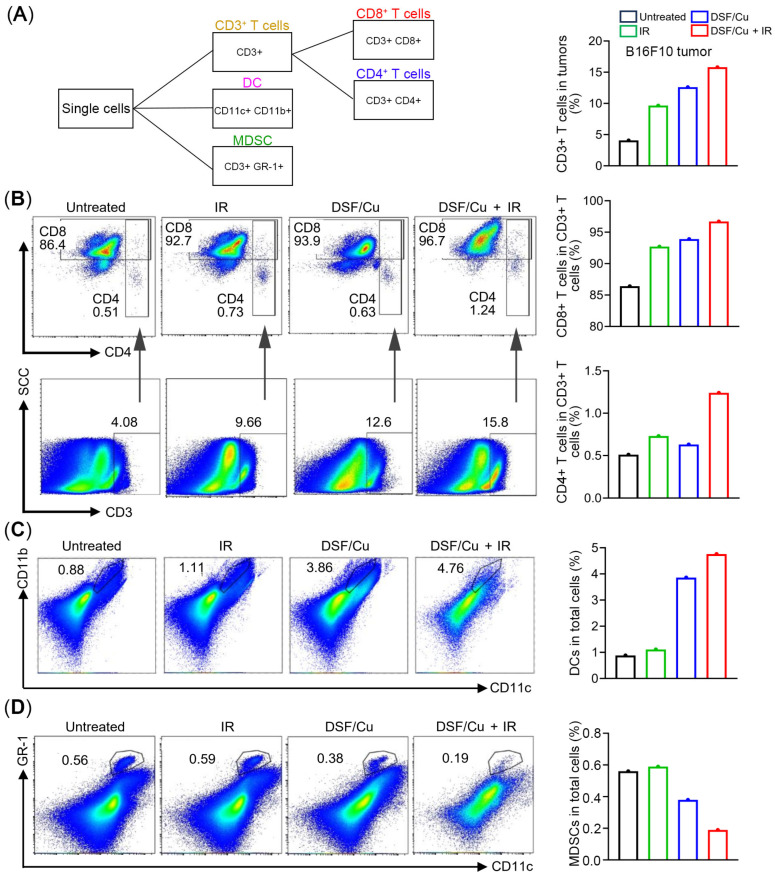
Flow cytometry analysis of tumor-infiltrating immune cells. Flow cytometry of tumor-infiltrating immune cells in B16F10 tumors following DSF/Cu and IR treatment. (**A**) The schematic of the gating strategy for CD8^+^ T cell, CD8^+^ T cell, DCs and MDSCs (**B**–**D**) The percentages of CD3^+^ T cells, CD3^+^ CD8^+^ T cells and CD3^+^ CD4^+^ T cells (**B**), DCs (CD11c^+^ CD11b^+^ cells (**C**) and MDSCs (CD11c^+^ and GR-1^+^ cells) (**D**) in B16F10 tumors are displayed, respectively. No p value was reported because tumor cells from mice within each treatment group were pooled into a single sample, as tumors in the DSF/Cu + IR group were too small to analyze individually.

Since ICD is associated with anti-tumor immune responses following treatment, we next analyzed single-cell suspensions of B16F10 tumors harvested from mice in different groups for tumor-infiltrating immune cell subsets using cell surface markers CD3, CD4, CD8, CD11b, CD11c or GR-1(Ly6g/Ly6c) (Figure 7A). Given that the tumors in DSF/Cu + IR-treated mice were too small to provide enough cells/per mouse for the analysis, cell suspensions from collected tumors within each experimental group were pooled into a single sample. The flow cytometry analysis indicated that both the IR-treated group and DSF/Cu-treated group exhibited an increased trend in infiltrating numbers of CD3+ T cells, CD8+ T cells and CD4+ T cells, compared to the control group. Moreover, the DSF/Cu + IR-treated group displayed more infiltrating numbers of CD3+ T cells, CD8+ T cells and CD4+ T cells than those in the DSF/Cu- or IR-treated groups (Figure 7B). Dendritic cells (DC; CD11b+ CD11c+ cells), the antigen-presenting cells that bridge innate and adaptive immunity, were increased in the tumors harvested from the DSF/Cu or IR-treated groups (Figure 7C). Meanwhile, compared with the control group, the infiltration of myeloid-derived suppressor cells (MDSCs) was decreased in the DSF/Cu treatment group and further decreased in the DSF/Cu + IR group (Figure 7C). Of note, the IR treatment did not induce the upregulation of DCs and the downregulation of MDSCs compared with the control group (Figure 7C,D). These findings suggest that DSF/Cu induces. These findings suggest that DSF/Cu induces ICD, leading to antitumor immune responses, and that DSF/Cu am-plifies the immunostimulatory effects of IR in melanoma.

## 3. Discussion

Disulfiram complexes with copper (DSF/Cu) have been shown to induce ICD of many tumor types including breast cancer, pancreatic cancer [25,37], and oral squamous cell carcinoma [38]. These findings prompted us to investigate whether combining DSF/Cu with IR presents a promising strategy to enhance ICD in melanoma cells, both in vitro and in vivo. This combined therapy aims to improve the immunogenicity of apoptotic melanoma cells, thereby augmenting the efficacy of immunotherapeutic interventions. Our study demonstrated that the combined DSF/Cu and IR treatment significantly increased levels of ICD markers, as indicated by increased CRT surface expression, elevated HMGB1 release, and decreased intracellular ATP levels—in MV3 and B16F10 melanoma cell lines compared to either treatment alone. These markers are indicative of a heightened immunogenic response against the apoptotic melanoma cells. In vivo studies revealed that tumors treated with DSF/Cu and IR exhibited suppressed growth and increased infiltration of immunostimulatory cells, including CD3^+^, CD4^+^, and CD8^+^ T cells, as well as DCs. Conversely, there was a reduction in immunosuppressive MDSCs. This shift suggests a more favorable TME for immune-mediated tumor eradication. Similar immune responses can also be observed in other animal models [39].

Although melanoma has shown responsiveness to immunotherapy, the overall cure rate remains low and responses are often not durable [40]. Cumulative clinical studies have reported that therapies enhancing tumor immunogenicity, such as ICD-inducing treatments combined with PD-1 checkpoint blockade [41,42,43], have shown improved efficacy in various cancers, including gastric, bladder, and breast cancers. Our study suggests that DSF/Cu and IR-induced ICD could potentially be integrated with ICI therapies to enhance treatment outcomes in melanoma.

The findings presented here align with our recent research demonstrating the role of DSF/Cu in enhancing ICD and its potential to transform tumors into in situ vaccines. In one study, we showed that DSF/Cu combined with IR induced a robust ICD in breast cancer cells, eliciting systemic anti-tumor immunity [37]. Additionally, we highlighted the capacity of stress-induced ICD in targeting cancer cells to reprogram chimeric antigen receptor (CAR) T cells, endowing them with early memory T cell characteristics, and to reshape the TME from “cold” to “hot”, thereby improving the efficacy of adoptive cell therapies [26].

ICD can induce adaptive immune responses by releasing adjuvant-like signals commonly recognized as DAMPs, including CRT, HMGB1, ATP, ANXA1, and type I IFN [44,45]. CRT is exposed on the plasma membrane surface of tumor cells and serves as an “eat me” signal by binding to LRP1 (CD91) on the surface of APCs, thereby activating innate immunity and adaptive immunity [46]. HMGB1, also known as amphoterin, is released from cancer cells undergoing ICD. Extracellular HMGB1 can bind multiple PRRs expressed by myeloid cells, encompassing advanced glycosylation end-product-specific receptor (AGER, best known as RAGE) and Toll-like receptor (TLR) [47]. During the ICD process, ATP is released in an autophagy-dependent manner and is exocytosed from the cell through the Annexin channel into ATP-containing vesicles. Furthermore, extracellular ATP acts as a “find-me” signal by engaging purinergic receptors, particularly P2RX7 and P2Y2, on dendritic cells and macrophages, thereby promoting myeloid cell recruitment and inflammasome activation at sites of active ICD [48]. Furthermore, ANXA1 is an important factor for DC homing, and the STING signaling pathway can activate the release of type I interferons, which activate immunity through binding to receptors on the surface of immune cells [45]. Notably, one limitation of this study is that the ANXA1 and STING/IFN signaling pathways were not detected.

Compelling evidence has demonstrated that inducing ICD is a promising strategy to enhance immunogenicity in tumors and to improve the efficacy of immunotherapy [49]. In addition, DSF/Cu significantly upregulates the expression of PD-L1 in HCC cells, thereby enhancing the response of HCC to PD-1 antibody therapy [50]. Previous studies indicated that DSF/Cu activates the cGAS-STING pathway via reactive oxygen species-induced DNA damage that improves antitumor response to PD-1 blockade [51]. Therefore, it can be speculated that the combination of DSF/Cu + IR can enhance the effect of the PD-1/PD-L1 blockade on antitumor activity.

This study has several unresolved issues that need further exploration. Firstly, the molecular mechanism of ICD induced by the combination of DSF/Cu and IR in melanoma remains unclear. Whether DSF/Cu + IR induces ICD through the cGAS/STING pathway and whether inhibition of aldehyde dehydrogenase 2 (ALDH2) activity plays an important role in DSF/Cu+ IR-induced ICD were not thoroughly explored. Secondly, the present study did not investigate the role of other patterns of cell death, such as cuproptosis, ferroptosis, apoptosis and necroptosis, in the DSF/Cu + IR-treated melanoma cells. In addition, samples were pooled due to low tumor cell numbers in the treatment group, which limited the statistical power of the in vivo immune cell analysis. Moreover, further investigation is required to determine whether DSF/Cu + IR can trigger chronic inflammation due to DAMP release. Furthermore, given the differences between the immune system of humans and mice [52], more in-depth experiments are needed to be conducted in the future, such as using a humanized mouse model to better simulate the human immune system. Furthermore, fractionated IR is standard-of-care for most solid tumors (e.g., 1.8–2 Gy per fraction, delivered daily over several weeks). Although a single 12-Gy dose shows robust biological activity in inducing immunogenic cell death (ICD) in vitro, its effects in vivo may differ from those of fractionated irradiation. To better bridge our experimental findings with clinical application, future studies should evaluate and compare the efficacy and safety of combining DSF/Cu with a single 12-Gy dose versus clinically relevant, fractionated radiotherapy regimens used for melanoma.

## 4. Materials and Methods

### 4.1. Cell Lines

Human melanoma MV3 cell line, generously provided by Prof. K. Zanker (Witten/Herdecke University, Germany), was cultured in RPMI 1640 (10-040-CV, Corning, NY, USA) supplemented with 10% fetal bovine calf serum (FBS, Gemini Bio, West Sacramento, CA, USA) and 1% (*v*/*v*) penicillin-streptomycin (Thermo Fisher Scientific, Waltham, MA, USA). Mouse melanoma B16F10 cell line, obtained from the American Type Culture Collection (ATCC), was maintained in DMEM (10-013-CV, Corning) with 10% FBS and 1% (*v*/*v*) penicillin–streptomycin. Both these cells have been authenticated using STR profiling and were maintained following the manufacturer’s instructions.

### 4.2. Chemical Reagents and X-Ray Machine

DSF (≥97.0%) and CuCl_2_ (99%) were acquired from Sigma-Aldrich (St. Louis, MO, USA). DSF was dissolved in Dimethyl Sulfoxide (DMSO, Sigma-Aldrich), and Cu was dissolved in sterile water. The X-RAD 320 Biological Irradiator (Precision X-ray, North Branford, CT, USA) was applied to in vitro and in vivo experiments in this study (once in vitro; fractionation in vivo). IR performed in our experiments was 12 Gy to induce ICD based on our previous studies, and calibration of x-ray measurement was carried out before each use according to the manufacturer’s instructions [25,26,37].

### 4.3. Cell Treatment DSF/Cu and/or IR In Vitro

Both B16F10 and MV3 cells were seeded in T75 flasks (Fisher Scientific, Pittsburgh, PA, USA) at a density of 1.0 × 10^6^ cells/flask with 10 mL complete medium. DSF was titrated with 0.1, 0.15, 0.2, 0.25, 0.3, 0.35 and 0.4 μM for exploring the appropriate concentration of DSF, while Cu was used at a fixed dose of 1 μM in vitro. This concentration range has been demonstrated to be safe and effective in our previous research [25,26,37]. Then cells were divided into 4 groups including (1) untreated, (2) treated with DSF/Cu, (3) treated with IR (12 Gy), and (4) treated with DSF/Cu combined with IR (12 Gy), respectively. After treatment for 24 h, the cell supernatants were collected and centrifuged at 400× *g* to remove cell debris, which were ready for enzyme-linked immunosorbent assay (ELISA) detection; the cells were digested by trypsin, centrifuged, and then subjected to subsequent flow cytometry analysis.

### 4.4. Cell Viability Assay

MV3 and B16F10 cells were seeded in 96-well plates at 5 × 10^3^ cells/well and incubated at 37 °C overnight. Subsequently, these cells were administered with DSF/Cu (DSF: 0–1 µM; Cu: 1 µM) for another 24 h. Cell viability was assessed by the 3-(4,5-dimethylthiazol-2-yl)-2,5-diphenyltetrazolium bromide (MTT) assay (Sigma-Aldrich) according to the manufacturer’s instructions, and the absorbance at 490 nm was examined using the Synergy 2 Multi-Detection Microplate Reader (BioTek, Winooski, VT, USA), and each sample had triplicate repetitions. The half-maximal inhibitory concentration (IC_50_) values of DSF/Cu were estimated through establishment of the dose–response curve.

### 4.5. Cell Apoptosis Assay

FITC Annexin V Apoptosis Detection Kit with 7-aminoactinomycin D (7-AAD, Biolegend, Dedham, MA, USA; catalog: 640922) was utilized to detect cell apoptosis, according to the manufacturer’s instructions. After treatment with DSF/Cu and/or IR for 24 h, the cells were collected and then stained with Annexin V and 7-AAD at 4 °C in the dark condition for 30 min. The apoptotic cells were subsequently analyzed by using the BD Accuri™ C6 Flow Cytometer (BD Bioscience, San Jose, CA, USA) according to the manufacturer’s instructions.

### 4.6. Flow Cytometric Analysis of Cell Surface Expression of CRT

Expression of CRT on cell surface was examined by employing a two-step method. After treatment with DSF/Cu and/or IR, the cells were collected and then stained with primary anti-human CRT monoclonal antibody TO-11 at 4 °C for 1 h [43], and then incubated with second antibody (Jackson immunoResearch Laboratories, Inc., Philadelphia, PA; cat: 115-116-146) in the dark at 4 °C for 1 h. The CRT expressions were subsequently assessed using BD Accuri™ C6 Flow Cytometer and FlowJo software (V10, TreeStar, San Carlos, CA, USA).

### 4.7. Detection of Intracellular ATP

ATP-sensitive fluorochrome quinacrine (Sigma-Aldrich) was used to stain melanoma cells for intracellular ATP [53]. The 5 × 10^4^ B16F10 and MV3 cells were incubated in 100 µL PBS supplemented with 10 µM quinacrine dihydrochloride at 37 °C for 1 h. After washing by PBS, these cells were resuspended in 100 µL PBS for flow analysis. Fluorescence of the melanoma cells was detected at 510–530 nm with excitation at 488 nm [54].

### 4.8. Quantitation of Extracellular HMGB1

The concentrations of extracellular HMGB1 were measured using HMGB1 ELISA Kit. Concentration of HMGB1 in B16F10 cell supernatants was detected by using mouse HMGB1 ELISA Kit (MBS722248, MyBioSource, San Diego, CA, USA), while HMGB1 in MV3 cell culture supernatants was measured by applying human HMGB1 ELISA Kit (ABIN511375, Antibodies online), according to the manufacturer’s instructions. HMGB1 levels were quantified at a wavelength of 450 nm in a Synergy 2 Multi-Detection Microplate Reader (BioTek).

### 4.9. Animals and Ethics Statement

In total, 22 female C57BL/6 mice were obtained from the Jackson Laboratory (Bar Harbor, ME, USA). We used female mice because of their resistant radio-sensitivity and less aggressive behavior. All mice were housed in autoclaved polysulfone individually ventilated cages (Allentown Caging, Allentown, NJ, USA) in a specific pathogen-free (SPF) environment, and each cage contained no more than five mice. Room lights were maintained on a 12:12 h light–dark cycle. Room temperature was maintained at 20 to 22 °C, food and water were provided ad libitum, and room humidity remained between 30% and 60%. All animal experiments were conducted in accordance with the protocol approved by the Institutional Animal Care and Use Committee (IACUC) at Massachusetts General Hospital (Protocol No. 2019N000025).

### 4.10. Therapy of B16F10 Melanoma-Bearing C57BL/6 Mice

At 11–18 weeks old, 20–24g C57BL/6 mice with complete immune systems were used to establish the B16F10 melanoma transplant tumor model. B16F10 cells/mouse (1 × 10^6^ cells in 100 µL DMEM) were injected subcutaneously in the right thighs of C57BL/6 mice. Tumor volume and body weight were monitored every two-days in blind method. Tumor volumes were calculated by the formula: volume = 0.5 × length × width^2^. Treatments of DSF/Cu and/or IR were initiated when the tumors had an approximate volume of 30 mm^3^ (day 1), such that the difference in mean tumor volumes was not statistically significant between each group. A total of 22 C57BL/6 mice were randomly divided into 4 groups (*n* ≥ 5 mice/group): (1) untreated group (*n* = 5): mice were left untreated. (2) DSF/Cu group (*n* = 6): mice were injected with 100 µL PBS containing DSF/Cu (DSF: 0.3 µM; Cu: 1 µM) intratumorally (i.t.) on day 1 and day 3. (3) IR (irradiation) group (*n* = 5): tumor was irradiated locally once (12 Gy) on day 2, while the remaining body was covered by a lead shield. The dose and route of DSF/Cu administration, as well as the specific irradiation (IR) regimen, were established in our previous studies [26,37]. (4) DSF/Cu+IR group (*n* = 6): mice were injected with DSF/Cu i.t. on day 1 and day 3, and tumor received IR on day 2. One cycle of above treatment is considered as one in situ cancer vaccination. Tumor size was measured every two days by using calipers, and the tumor volume was calculated through the formula: volume = width^2^ × length/2. After 3 cycles of vaccination, the mice were euthanized via CO_2_ inhalation when tumor volume reached 2000 mm^3^, and their tumors were collected for flow cytometry analysis. All 4 groups were blinded to investigators.

### 4.11. Preparation of a Single-Cell Suspension of Mouse Melanoma Tumor Tissue for Flow Cytometry Analyses

Non-necrotic primary tumor specimens from each group were collected after sacrifice, disrupted into 3 × 3 mm pieces, and further dissociated by Collagenase IV (1 mg/mL) (Worthington Biochemical Corp., Lakewood, NJ, USA) for 1h at 37 °C. A single-cell suspension was obtained by filtering the digested tissue through a 70-μm cell strainer, followed by centrifugation and centrifugation at 450× *g* for 15 min. The cell pellets were resuspended with 5 mL RBC lysis buffer (A1049201, ThermoFisher Scientific) and incubated for 5 min at room temperature, and 5 mL culture medium was added to stop the lysis. After washing, the cell pellets were resuspended in PBS.

### 4.12. Characterization of Tumor-Infiltrating Immune Cells by Flow Cytometry Analysis

The single-cell suspension samples were stained with multiple conjugated antibodies recognizing mouse CD3/CD4/CD8/CD11b/CD11c/GR-1 markers (Table S1) at 4 °C for 1 h in the dark with appropriate isotype control antibodies according to the manufacturer’s instructions. Flow cytometric analyses were performed by using BD LSRFortessa X-20 (BD Biosciences) following the manufacturer’s instructions. FlowJo V10 (TreeStar, San Carlos, CA, USA) was used to analyze the flow data.

### 4.13. Statistical Analysis

Statistical analysis was performed by GraphPad Prism 9.0, and all results were presented as means ± standard deviation (SD). Student’s *t*-test (unpaired) was used when two groups were compared. Normality was confirmed via visual inspection of Q-Q plots and the Shapiro–Wilk test. Each experiment was repeated at least three times. *p* value < 0.05 was considered statistically significant.

## 5. Conclusions

In summary, the integration of DSF/Cu and IR to induce ICD presents a promising strategy to overcome the inherent resistance of melanoma to conventional therapies. By enhancing tumor immunogenicity and modulating the TME, this approach could improve the effectiveness of existing immunotherapies, including checkpoint inhibitors and adoptive cell therapies. Future clinical studies are warranted to validate these findings and explore the translational potential of this combination therapy for melanoma patients.

## Data Availability

The original data supporting the conclusions of our article will be available from the corresponding author upon reasonable request.

## Supplementary Materials

The following supporting information can be downloaded at: https://www.mdpi.com/article/10.3390/ijms27020980/s1.
