# Supplementary material for: Disulfiram/Copper Combined with Irradiation Induces Immunogenic Cell Death in Melanoma
Source: Int J Mol Sci. 2026 Jan 19;27(2):980. doi: 10.3390/ijms27020980 (PMC12842420; doi:10.3390/ijms27020980)
Supplement: Supplementary file 1 [file ijms-27-00980-s001.zip › ijms-4033157-supplementary.pdf]

Table S1. Antibodies used of analysis of tumor-infiltrating immune cell surface antigens

**Antibody**	**Fluorophore**	**Suppliers**	**Catalogue number**	**Clone**
CD3 (APC/Cyanine7 anti-mouse CD3)	APC/Cyanine7	Biolegend	100222	17A2
CD4 (Alexa Fluor® 488 anti-mouse CD4)	Alexa Fluor® 488	Biolegend	100529	RM4-5
CD8 (BD Pharmingen™ PE-Cy™7 Rat Anti-Mouse CD8a)	PE-Cy™7	BD Biosciences	552877	53-6.7
CD11b (BD Horizon™ BV650 Rat Anti-CD11b)	BV650	BD Biosciences	563402	M1/70
CD11c (Brilliant Violet 421™ anti-mouse CD11c)	Brilliant Violet 421™	Biolegend	117329	N418
GR-1 (Ly6g/Ly6c) (BD Pharmingen™ APC Rat Anti-Mouse Ly-6G and Ly-6C)	APC	BD Biosciences	553129	RB6-8C5
